# Monoradicular Primary Maxillary Left Second Molar: A Rare Root Morphology and Review of the Literature

**DOI:** 10.1155/crid/1034638

**Published:** 2026-05-15

**Authors:** Basim Almulhim

**Affiliations:** ^1^ Department of Orthodontics and Pediatric Dentistry, College of Dentistry, Qassim University, Buraydah, Saudi Arabia, qu.edu.sa

**Keywords:** conical, pyramidal, root anomaly, single, tubular

## Abstract

Understanding the anatomical variations between primary and permanent dentitions and familiarity with the internal and external root morphology are essential for dental practitioners to distinguish root morphological anomalies. A search in dental literature reveals the occurrence of monoradicular molars in primary dentition; especially the monoradicular maxillary primary second molar is extremely rare, returning only five case reports. The present case of a 5‐year‐and‐7‐month‐old girl presented with experience of severe pain in her upper left back area. After taking the medical and dental histories and conducting a clinical and radiographic examination, the examinations showed a healthy child, carious teeth, a localised abscess and a rare root morphology (monoradicular maxillary primary second molar), which has never been previously reported in the Saudi population. The treatment was performed using quadrant dentistry, which involved a pulpotomy and a stainless steel crown, extraction and a reverse band and loop, whilst keeping the child under regular follow‐up. The aim of this report is to assist dental practitioners, particularly dentists, in recognising this root anomaly that is a rare monoradicular maxillary left second molar so as to minimise the associated complications. In addition, this paper summarises similar cases in the dental literature. Achieving a high level of practical dental skills and accurate dental diagnoses promotes the standard of dental treatment, in turn leading to successful endodontic results.

## 1. Introduction

Recognising and understanding the implications of uncommon anatomical variations between primary and permanent dentition, especially the internal and external root morphology of teeth, are essential skills for dental practitioners. [1]. Usually, primary dentition shows fewer common dental anomalies than permanent dentition, particularly in relation to number, shape and size [2]. Maxillary second molars are commonly three‐rooted; however, rarely, these teeth are monoradicular. This extremely rare condition is more prevalent in females than males [1, 3, 4]. Dental caries frequently affects both dentitions, but in primary dentition the pattern of progression of caries differs from that in permanent dentitions, spreading extensively and rapidly, often involving the pulp within short periods [1].

Furthermore, any dental aberration in different stages of tooth development and formation will lead to variation in tooth structure [5]. In primary dentition, formation of the root is completed within 2–3 years of eruption of the tooth [5]. Moreover, root abnormalities are exceedingly uncommon; where they occur, the majority are associated with hypercementosis, dilacerations and concrescence [6]. Presently, the aetiology of the unusual monoradicular root anomaly is unknown. However, environmental, genetic and biological factors may contribute to the aetiology of these root anomalies. These factors may lead to abnormal root structures forming during the root formation period, which may correlate to the invagination of derivatives of the enamel organ [7]. Some genetic or biological factors, such as homeobox (HOX), may contribute to root development and formation. Any change in the maturation of these genes may affect and alter root formation and development [3]. Moreover, it is essential that dental practitioners have a robust understanding of normal root morphology so as to identify related anomalies. By enhancing dental practical skills and paying special attention during dental diagnosis, dental practitioners can deliver high‐quality dental treatment, which will lead to successful endodontic results [7]. A search of the literature found only five case studies that focus on monoradicular maxillary left second molars (Table 1). The present case is the sixth reported monoradicular maxillary second molar globally and the first among the Saudi population. The purpose of this paper is to enhance the knowledge of dentists and raise their awareness of the phenomenon of abnormal root morphology, such as monoradicular maxillary left second molars.

**Table 1:** This table provides a review of cases of monoradicular maxillary second primary molars solely through the previous dental literature.

Author	Year	Number of cases	Gender	Diagnostic tools	Inheritance pattern	Description
Ackerman et al. [8]	1973	Study in 20 family members	Males/females, boys/girls	Periapical radiographs + orthopantomographs	The possibility of an autosomal dominant gene with variable penetrance or a codominant autosomal gene cannot be excluded. There was no history of consanguinity.	There are noticeable monoradicular primary molars and permanent monoradicular first molars among family members.
		One case	10 years/boy			
Holan and Chosack [9]	1991	Two cases	9 years/girl	Periapical radiographs + orthopantomographs	Parents were close relatives, which possibly increases their children′s homozygosity. No syndromes were noticed. They suggested that the inheritance pattern is autosomal recessive.	There are both monoradicular primary molars and monoradicular permanent first molars for both cases.
			8 years/boy			
Nguyen et al. [10]	1996	Three cases	40 years/male	Orthopantomographs	They suggest the trait in this family is inherited in an autosomal dominant pattern. No systematic disease was noticed.	There are noticeable monoradicular primary molars in the youngest child and permanent monoradicular molars among other members.
			12 years/girl			
			8 years/girl			
Ballal et al. [11]	2006	One case	40 years/female	Periapical radiograph + spiral computer tomograph	Not specified	There is a retained monoradicular right maxillary primary second molar.
Kumar et al. [12]	2010	One case	11 years/boy	Orthopantomograph	They suggest variations of a single heritable trait. No syndromes were noticed.	There are both monoradicular primary molars, as well as monoradicular permanent molars with noted nonsyndromic oligodontia.
Present case	2025	One case	8 years/girl	Periapical radiograph	No syndromes or family history was noticed.	There is a monoradicular left maxillary primary second molar.

## 2. Case Presentation

A private dental care clinic in the Qassim region of Saudi Arabia received a referral for a 5‐year‐and‐7‐month‐old Saudi girl as an emergency case. The mother, who attended the clinic with her daughter, reported that the child had been experiencing severe pain in her upper left back area at night for the past week. The mother attempted to visit multiple dental clinics but was unsuccessful due to her child previously having a negative dental experience that made her reluctant to accept dental treatment (Frankel Score II). The child was taking medication (5 mL of ibuprofen and 4 mL of amoxicillin) that had been prescribed by a general dentist. The child′s medical history was reviewed, and she showed no known medical illnesses, allergies or previous hospitalisations. An intraoral examination revealed the child′s early mixed dentition to include a Class I canine relationship, a flush terminal molar relationship, spaced teeth, midline coincidence and poor oral hygiene. The clinical dental exam revealed multiple caries lesions and a localised abscess in the upper left primary second molar. A radiographic examination was undertaken, in which a periapical x‐ray revealed multiple carious lesions in the upper and lower teeth. It was proposed that the child undergo a full dental rehabilitation under general anaesthesia. However, the mother was particularly concerned about the cost of general anaesthesia and expressed some concerns about the risks of related complications. Furthermore, the mother expressed her desire to treat her child in a dental setting. Different behavioural techniques, including nonpharmacological and pharmaceutical techniques, were used to calm the child. After discussing comprehensive treatment options with the mother, the treatment proceeded using nitrous oxide to sedate the child, an approach that was accepted by the mother. Moreover, whilst under the effects of the nitrous oxide, emergency dental treatment was carried out. Local anaesthesia was administered as infiltration to buccal and palatal sites. The dental treatment included a pulpotomy and a stainless steel crown on the upper left primary first molar and extraction of the upper left primary second molar with the use of a reverse band and loop. Following the dental procedure, 100% oxygen was administered to the girl for 5 min without any complications. Postoperative instructions were given to the girl′s mother, and the child was discharged from the dental clinic. The child was kept under review with regular follow‐ups. Figures 1, 2 and 3 show the intraoral preoperative periapical radiograph, along with clinical and digital images of the extracted teeth.

**Figure 1:**
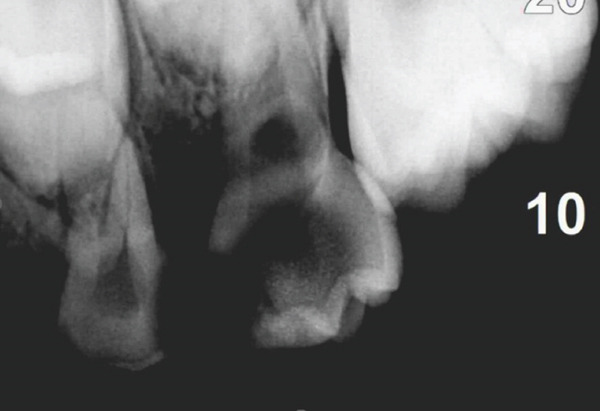
This figure shows the preoperative periapical dental radiograph for the maxillary left quadrant. The image reveals a monoradicular structure in the maxillary second molar, along with deep caries in both the primary first and second molars, as well as a partially erupted maxillary left permanent first molar.

**Figure 2:**
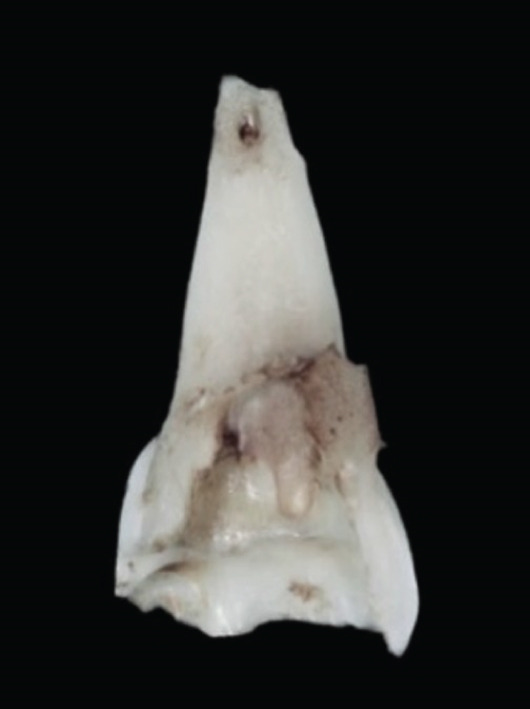
This figure shows the palatal view of the tooth after extracting the maxillary left second primary molar.

**Figure 3:**
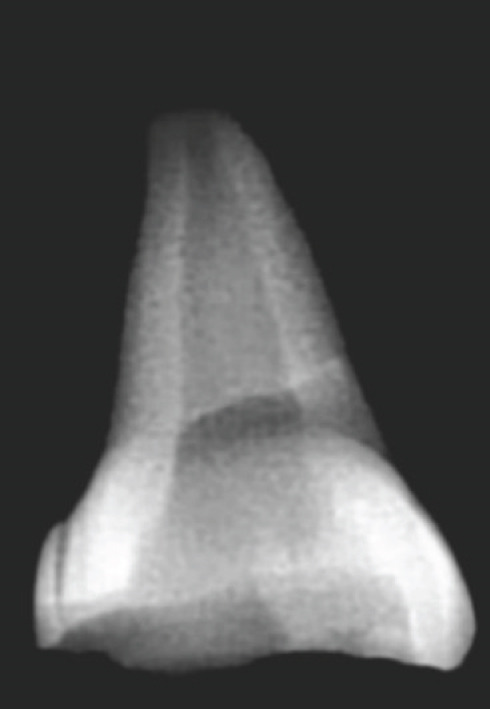
This figure is a digital radiographic image of the extracted upper left second primary molar.

## 3. Discussion

It is not uncommon for permanent third molars to exhibit some variation in the morphology of their roots and root canal, especially monoradicular abnormalities. However, according to the extant dental literature, anomalies like these are rarely detected in primary dentition. Vertucci′s system classifies root canals, categorising the monoradicular tooth as Type 1, as it has a single root canal; it is a rare occurrence in primary molars [13]. The first case reported in dental literature was that of a 10‐year‐old child who had single roots in all primary maxillary and mandibular molars, as well as single roots in all first permanent maxillary and mandibular molars [8].

The previously reported cases showed occurrence variations for this root anomaly. The dental literature typically observes this root anomaly in both genders [8–12]. A girl child exhibited the present case, aligning with previous reports in dental literature [8–11]. However, other cases were noticed among boys [8–10, 12]. Furthermore, the present case was shown in a healthy child with no syndrome or any related family history. Kumar et al. reported similar observations, noting variations of a single heritable trait without any associated syndromes [12]. However, many case reports are linked to genetics [8–10]. Ackerman et al. link to the possibility of an autosomal dominant gene with variable penetrance or a codominant autosomal gene that cannot be excluded and mention no history of consanguinity [8]. Robbins and Keene proposed an autosomal dominant inheritance pattern [14]. Another case was reported by Holan and Chosack, who noted that the parents were close relatives, which may increase their children′s homozygosity; however, no syndromes were observed, and they suggested that the inheritance pattern of the monoradicular shape in both permanent and primary dentitions is autosomal recessive [9]. Nguyen et al. suggested the trait in this family is inherited in an autosomal dominant pattern with no systematic disease noticed [10]. Furthermore, this root anomaly may occur unilaterally or bilaterally in presentation. In the present case, whether this unilateral variation is truly isolated or possibly bilateral remains unknown, limited to the unilateral x‐ray that was taken. Unilateral presentation previously reported by Ballal et al. [11]. However, Holan and Chosack and Kumar et al. reported there are both monoradicular primary molars and monoradicular permanent first molars for both cases [9, 12]. These variations in occurrence may be linked to several factors, including genetic, environmental factors or multifactorial inheritance, which can influence the development of root morphology in the late‐bell stage, potentially leading to differences in the number of roots present.

Usually, the normal root development begins after crown formation during the late bell stage. The Hertwig′s epithelial root sheath (HERS), a bilayered structure derived from the inner and outer dental epithelia, grows apically to determine root number, shape and length. In multirooted teeth, HERS develops epithelial projections that divide the single root opening into two or three separate foramina. Failure of this division results in a single conical root. However, the main aetiology of the unusual monoradicular root anomaly is unknown. Many factors may lead to abnormal root structures forming during the root formation period, which may correlate to the invagination of derivatives of the enamel organ, such as environmental, genetic and biological factors. Some genetic or biological factors, such as HOX genes, may contribute to root development and formation. Any change in the maturation of these genes may affect and alter root formation and development. Environmental factors that could theoretically interfere with HERS, including dental trauma, radiation and chemotherapy, may contribute to the formation of this anomaly, as these factors can disrupt the normal pathways involved in root development and lead to abnormal root morphology [3, 7, 15]. In the present case, there is no identifiable main cause, whether it is environmental or genetic or related to a syndrome supporting a sporadic developmental anomaly. The absence of similar findings in family history further suggests a nonhereditary aetiology.

There are several terms that have been used to describe the monoradicular molars in the primary dentition, such as ‘pyramidal’, ‘taurodontism’, ‘conical’ and ‘fused’ [12, 15]. In most cases, this root anomaly′s remaining asymptomatic nature is accidentally detectable in a dental clinic during a routine examination and does not usually require intervention. Typically, diagnosing such root anomalies is achieved in normal dental settings from two‐dimensional dental radiographs, such as periapical radiographs (bisecting or parallel technique). Dental practitioners sometimes prefer to take dental radiographs from different horizontal angles, such as those with 20° of distal and mesial angulation; these can be advantageous for making reliable diagnoses, especially in young children [16]. Furthermore, for more accurate diagnosis of root anomalies, three‐dimensional dental radiographs, such as cone beam computed tomography (CBCT) or spiral computer tomographs (SCT) or helical computer tomographs (HCT), are helpful [17, 18]. Excessive bleeding during dental procedures, particularly pulpotomy or pulpectomy, especially after complete pulp expiration, typically signals severe pulp inflammation or iatrogenic perforation. This condition can stem from a lack of dental experience or inadequate dental knowledge of root variation in particular [19]. Usually, in the multirooted molar, the pulpal floor contains distinct orifices leading to separate root canals. However, in such rare cases of a monoradicular tooth, the pulpal floor anatomy is altered, and specifically, there is no furcation and no multiple canal orifices. This altered morphology may mislead a clinician who is accustomed to the standard anatomy, causing them to inadvertently search for nonexistent other canals. Consequently, excessive bleeding encountered during pulpectomy of a monoradicular primary molar should raise immediate suspicion of iatrogenic perforation rather than pulp inflammation alone. Clinicians are advised to confirm root and canal number radiographically before initiating endodontic treatment in suspected anatomical variants. It is essential to raise and develop the practical dental skills of dental practitioners, especially the dentists, including their ability to deliver accurate diagnoses, which is fundamental to delivering high‐quality dental treatment. There are several clinical implications for a notable anatomical abnormality of a single root in primary molars, with emphasis on the importance of a clinical approach starting with a dental diagnosis to deliver high‐quality dental treatment. The first challenge is to confirm a dental diagnosis, which is usually based upon a regular dental radiograph, as a clinical examination alone is an inadequate method of making the diagnosis. The second challenge is that two‐dimensional dental radiographs provide only two aspects of the root′s anatomy. CBCT is helpful for more accurately confirming the diagnosis, analysing the shape of the root, and numbering the canal. However, CBCT is unsuitable for children because it is a high‐radiation imaging technique, which raises concerns about the potential long‐term effects of radiation exposure on developing tissues. The third challenge is that these teeth are very significant in root canal treatment for children because they often have multiple curved roots, which may complicate the dental treatment. A lack of understanding of the anatomy can lead to some complications, such as perforation. Fourthly, the conical, single cylindrical shape seen in monoradicular molars is usually observed for anterior teeth, and normally the movement used during dental extraction is rotational. However, for extraction of posterior teeth, the normal movement is buccolingual, as that expands the socket whilst avoiding root fracture. Using this manoeuvre for a single root can increase the risk of fracturing the root. By enhancing dental practitioners′ knowledge of normal root anatomy and related anomalies, it promotes the likelihood of excellent outcomes. Usually, the variations in root morphology will lead to some complications during dental treatment if they are not notable. For dentists, prioritising dental radiography is essential. Paying special attention to root variations during the normal growth and development of primary teeth could help prevent or reduce future complications. Only a periapical radiograph was taken on the affected area; consider this the main limitation of this report. No other dental radiograph, such as an orthopantomogram, was performed due to the child′s apprehensive behaviour and the mother′s concerns about radiation exposure for her child. Therefore, the morphology of the primary maxillary right second molar as a contralateral could not be assessed. Whether this unilateral variation is truly isolated or possibly bilateral remains unknown. Future case reports are encouraged to include bilateral imaging when indicated and clinically feasible.

## 4. Conclusions

The aim of presenting this case of a unique root anomaly is to help to guide dental practitioners to achieve favourable treatment outcomes for their patients with minimal complications. The report′s goal is to increase dental practitioners′ knowledge by giving a comprehensive picture of abnormal root morphology and emphasise the importance of prioritising dental radiographs for regular dental care. Clinicians should maintain a high index of suspicion for anatomical variants when a two‐dimensional radiograph shows lack of root furcation, and using the appropriate behavioural technique in a dental setting, such as a nonpharmacological or a pharmacological approach like nitrous oxide, particularly in anxious children, can be effective when managed with the right behavioural techniques instead of general anaesthesia. Furthermore, to deliver high‐quality dental treatment, it is essential that dentists develop their practical dental skills, starting with making accurate dental diagnoses.

**Nomenclature**
CBCT: cone beam computed tomography
SCT: spiral computer tomographs
HCT: helical computer tomographs

## Author Contributions

B.A.: conceptualisation, data curation, formal analysis, investigation, methodology, project administration, resources, visualisation, writing the original draft and writing review and editing.

## Funding

No funding was received for this manuscript.

## Disclosure

I have read and approved the final version of the manuscript. As the corresponding author and manuscript guarantor, I had full access to all the data in this study and take complete responsibility for the integrity of the data and the accuracy of the data analysis.

## Ethics Statement

The author has nothing to report.

## Consent

This reporting case follows the CARE guidelines, and written informed consent was obtained from the patient′s guardian. No data on the paper reveals the patient′s identity.

## Conflicts of Interest

The author declares no conflicts of interest.

## Data Availability

The author confirms that the data supporting the findings of this study are available within the article.
